# Inhibition of EZH2 mitigates peritoneal fibrosis and lipid precipitation in peritoneal mesothelial cells mediated by klotho

**DOI:** 10.1080/0886022X.2022.2149411

**Published:** 2023-02-01

**Authors:** Qinglian Wang, Jingshu Sun, Rong Wang, Jing Sun

**Affiliations:** a Department of Nephrology, Shandong Provincial Hospital Affiliated to Shandong First Medical University, Jinan, Shandong, China; b Department of Nephrology, Shandong Provincial Hospital Affiliated to Shandong University, Jinan, Shandong, China; c Department of Nephrology, Weifang People’s Hospital, Weifang, Shandong, China

**Keywords:** Peritoneal fibrosis, peritoneal dialysis, EZH2, klotho, lipid accumulation

## Abstract

**Background:**

Peritoneal fibrosis caused by long-term peritoneal dialysis (PD) is the main reason why patients withdraw from PD treatment. Lipid accumulation in the peritoneum was shown to participate in fibrosis, and klotho is a molecule involved in lipid metabolism. GSK343 (enhancer of zeste 2 polycomb repressive complex 2 subunit (EZH2) inhibitor) has been verified to inhibit epithelial mesenchymal transdifferentiation (EMT) and peritoneal fibrosis, but its related mechanism remains unclear. This study aimed to investigate whether lipid accumulation was involved in the effect of GSK343 and its related mechanism.

**Materials and Methods:**

First, the expression of EZH2, klotho and EMT indices in human peritoneal mesothelial cells (HMrSV5) incubated with high glucose (HG) levels was detected. After EZH2 was inhibited by GSK343, Western blot (WB), wound healing and Transwell assays were used to explore the effect of GSK343. EZH2 and klotho expression was also detected. Oil red O and Nile red staining and triglyceride (TG) detection kits were used to detect lipid accumulation. A rescue experiment with small interfering RNA specific for klotho (si-klotho) on the basis of GSK343 was also conducted to verify that GSK343 exerted its effect *via* klotho. In *in vivo* experiments, rats were administered GSK343, and the related index was assessed.

**Results:**

In our study, we revealed that the expression of EZH2 was significantly upregulated and klotho was significantly downregulated in HMrSV5 cells induced by high glucose. With the aid of GSK343, we found that lipid deposition caused by HG was significantly decreased. In addition, EMT and fibrosis were also significantly alleviated. Moreover, GSK343 could also restore the downregulation of klotho. To further verify whether klotho mediated the effect of EZH2, a rescue experiment with si-klotho was also conducted. The results showed that si-klotho could counteract the protective effect of GSK343 on high glucose-induced lipid accumulation and fibrosis. *In vivo* experiments also revealed that GSK343 could relieve peritoneal fibrosis, lipid deposition and EMT by mitigating EZH2 and restoring klotho expression.

**Conclusions:**

Combining these findings, we found that EZH2 regulated lipid deposition, peritoneal fibrosis, and EMT mediated by klotho. To our knowledge, this is the first study to demonstrate the effect of the EZH2-klotho interaction on peritoneal fibrosis. Hence, EZH2 and klotho could act as potential targets for the treatment of peritoneal fibrosis.

## 1. Introduction

Peritoneal dialysis (PD) is a safe and convenient life-sustaining renal replacement treatment for patients with end-stage renal disease (ESRD) worldwide. According to the latest data, PD patients account for 11% of the total dialysis population [1–3]. In many countries, not only are the outcomes of patients with PD comparable to or even better than those who receive hemodialysis, but PD is also more cost effective. The advantages of PD are obvious, as it provides hemodynamic stability, retention of residual renal function (RRF) and a higher quality of life [4]. PD uses the peritoneum as a semipermeable membrane to remove toxins and metabolic waste to improve water, electrolyte metabolism and acid-base balance disorders. However, exposure to nonphysiological peritoneal dialysis fluid of the peritoneal membrane can lead to peritoneal dysfunction and eventually force patients to withdraw from PD. Pathologically, peritoneal tissue is characterized by epithelial mesenchymal transformation (EMT) of peritoneal mesothelial cells, cell shedding, and extracellular matrix accumulation [5]. Numerous studies have suggested that various stimulants, including high glucose, glucose degradation products (GDPs), advanced glycation end-products (AGEs), transforming growth factor β (TGF β) and low pH, are involved in peritoneal fibrosis and the triggering of pathological processes [6]. Many researches focused on TGF β and revealed that it plays a central role in peritoneal fibrosis. In our previous researches we also conducted. Therefore, in next step we will further clarify the pathogenesis induced by high glucose will provide new ideas for future treatment strategies.

In our previous studies, we found that high glucose could account for these pathological changes [7]. However, the detailed mechanisms are still unclear. Glucose metabolism is always closely related to lipid metabolism, which is called glucolipid metabolism. Moreover, chronic high glucose exposure eventually causes lipid accumulation in nonfatty tissues (lipotoxicity), such as the liver and skeletal muscle [8,9]. Studies also [10,11] showed that lipid deposition in kidneys [12] and in podocytes was increased under high glucose conditions. Reports have also suggested that abnormal lipid homeostasis may result in the pathogenesis of organ fibrosis, such as vascular sclerosis [13], renal fibrosis [14], cardiac fibrosis [15] and nonalcoholic fatty liver disease [16]. Reducing lipid accumulation can effectively alleviate fibrosis and extracellular matrix (ECM) deposition through various pathways in multiple organs. Interestingly, Chang et al. found that lipid-lowering agents (e.g., statins) inhibited ECM accumulation in high glucose-treated peritoneal mesothelial cells (PMCs) and peritoneal dialysis fluid (PDF)-stimulated rats *via* the mevalonate pathway [17]. Recently, Liu found that rapamycin exhibited a clear protective effect on PDF-induced peritoneal fibrosis by improving the disruption of intracellular lipid homeostasis [18].

Enhancer of zeste homolog 2 (EZH2) is a histone lysine methyltransferase that catalyzes the methylation of histones. Histone modification leads to altered chromatin structure and affects the accessibility of transcription factor DNA promoters. Currently, research on EZH2 is mainly focused on its role in tumorigenesis, and inhibition of EZH2 activity has become a new strategy for antitumor therapy. In addition, studies also revealed that EZH2 had a close relationship with hepatic fibrosis, kidney fibrosis and bone marrow fibrosis. In our previous research, we found that GSK343 (an inhibitor of EZH2) exhibited protective effects in high glucose-treated human peritoneal mesothelial cells (HPMCs) [7]. However, whether it plays a role by reducing lipid deposition is still unclear. In the present study, we further explored the related mechanism in peritoneal fibrosis.

Klotho (KL) protein was originally discovered in mice and identified as an antiaging gene with both membrane-bound and soluble forms [19]. Evidence has shown that deficiency renders multiple organs more susceptible to fibrosis, such as the kidney [20], heart [21], and lung [22], a common feature of aging [23]. Previous findings have indicated that the secreted form of klotho protein can function as a circulating hormone to exert multiple physiological effects on target organs [24]. Furthermore, recent discoveries showed that klotho could inhibit ROS production and scavenge excessive ROS in mouse tubular epithelial cells [25]. Huang et al. found that klotho antagonizes pulmonary fibrosis by suppressing pulmonary fibroblast activation, migration, and extracellular matrix production, demonstrating a potential therapeutic option for idiopathic pulmonary fibrosis [26]. Most recently, Kadoya’s team reported that klotho protected the peritoneal membrane from peritoneal fibrosis in transgenic-PD mice through attenuation of the Wnt/β-catenin signaling pathway [27]. However, the specific mechanism involved is still unclear. Studies have also clarified that klotho protein participates in lipid metabolism in numerous disease models. Therefore, this raises the question of whether klotho participates in peritoneal fibrosis by regulating lipid metabolism.

Based on the above findings, we hypothesized that GSK343 could attenuate peritoneal dialysis-related peritoneal fibrosis by reducing lipid deposition through klotho protein regulation. To test this hypothesis, we performed both *in vitro* and *in vivo* experiments to determine whether GSK343 exerts protective effects on lipotoxicity *via* klotho protein and aimed to clarify the relevant mechanisms involved in this process.

## 2. Materials and methods

### 2.1. Chemicals and reagents

A 10 mM stock solution of EZH2 inhibitor (HY-13500, MedChemExpress Bio-Technology, Shanghai, China) was purchased and stored at −80 °C. The dilutions for the working solution did not exceed 0.1% DMSO in the medium. Dulbecco’s modified Eagle’s medium (DMEM F12/1:1), fetal bovine serum (FBS), and trypsin/EDTA were purchased from HyClone (Logan, UT, USA). Nile red (N8440) and Oil red O stains (G1262) were purchased from Solarbio Company. Klotho siRNA (sc-43883) was purchased from Santa Cruz Biotechnology. A tissue triglyceride assay kit (E1013) was purchased from Pplygen Company (Beijing, China). The primary antibodies were as follows: anti-EZH2 (ab191080, Abcam, Cambridge, MA, USA) for WB, as well as anti-E-cadherin (ab231303, Abcam, USA), anti-N-cadherin (ab76011, Abcam, USA), anti-vimentin (ab92547, Abcam, USA), anti-FN (ab2413, Abcam, USA), anti-klotho (ab181373, Abcam, USA) and anti-GAPDH antibodies. Horseradish peroxidase (HRP)-conjugated goat anti-rabbit and goat anti-mouse antibodies were provided by Beyotime Biotechnology (Shanghai, China). All other chemicals were of reagent grade and endotoxin free.

### 2.2. Cell culture

HMrSV5 cells were conditional cultured human peritoneal mesothelial cells. They were obtained from Professor Xueqing Yu. Cells were cultured in DMEM F12/1:1 medium supplemented with 10% fetal bovine serum, streptomycin (100 μg/mL), and penicillin (100 units/mL) at 37 °C in a 95% air/5% CO_2_ humidified atmosphere. When the cells reached confluence, they were rendered quiescent by incubation with DMEM F12/1:1 containing 1% serum [7]. The quiescent cells were then treated with high glucose (1.25%, 2.5%), mannitol (1.25%) or high glucose plus GSK343 (5, 10 μM) [7] for various durations as indicated. GSK343 was added 60 min before the addition of high glucose. After the cells were stimulated as needed, they were collected in accordance with the protocols for different experiments. For Western blotting, cells were scraped from the plate with lysis buffer. For the triglyceride assessment assay, cells were digested with trypsin and collected in EP tubes.

### 2.3. Western blot analysis

Western blotting was carried out as described previously [7]. Briefly, total protein (20 μg) was separated in 10% SDS–PAGE gels and transferred to polyvinylidene fluoride (PVDF) membranes. The membranes were then blocked with 5% nonfat milk for 1 h at room temperature and incubated at 4 °C overnight with the following primary antibodies. After incubation, the membranes were washed and reprobed with HRP-conjugated anti-mouse IgG and anti-rabbit IgG secondary antibodies for 1 h at room temperature. Protein bands were detected using an ECL system and a Bio-Rad electrophoresis image analyzer (Bio-Rad, Hercules, CA, USA).

### 2.4. Oil red O staining

Human peritoneal mesothelial cells were seeded on glass coverslips that had already been placed in a 24-well plate. The density of HPMCs was maintained at 5 × 10^4^ per well. After the cells were treated under different conditions, the glass coverslips were fixed with 4% paraformaldehyde for 30 min and washed with PBS. Then, the coverslips were stained with Oil Red O working solution for 15 min at room temperature. They were then washed with 70% alcohol and counterstained with HE for 2 min. Finally, the coverslips were sealed with glycerogelatin and imaged in randomly selected fields with OLYMPUS CellSens.

### 2.5. Nile red staining

HPMCs were seeded in 24-well plates at a density of 5 × 10^4^ cells per well. After treatment with different conditions and times, Nile Red was used for neutral lipid staining. Cells were incubated with 1 μM Nile Red solution in a 37 °C incubator for 10 min and observed under a fluorescence microscope at EX/EM = 530/635 nm. Images were obtained in randomly selected fields with an OLYMPUS CellSens.

### 2.6. Triglyceride content assessment

Triglyceride (TG) content was assessed by a tissue triglyceride assay kit (E1013, Pplygen company of Beijing, China). Procedures were performed according to the manufacturer’s instructions as detailed. Briefly, cells were digested with trypsin and collected in EP tubes. Moderate lysis buffer (provided) was added. Then, the lysis buffer was separated into two parts: one part was used to detect the protein concentration, and the other part was used to detect the TG content. According to the standard substance (provided) OD value, a fitting curve was established. Then, the TG content of our expected samples was calculated. The OD value was detected by a microplate reader (SpectraMax M2, Molecular Devices, USA), and the TG content was normalized per mg protein concentration.

### 2.7. Transfection of si-klotho

Small interfering RNA (siRNA) targeting klotho (si-klotho, sc-43883) was designed and purchased from Santa Cruz Biotechnology. When HPMCs reached approximately 60–70% confluency, they were transfected with Opti-MEM medium containing si-klotho/NC and Lipofectamine™ 2000 for 6 h. The medium was changed, and the cells were grown further with fresh DMEM/F-12 medium for another 24 h. The transfected HMrSV5 cells were then treated with high glucose plus GSK343 (10 μM).

### 2.8. Wound healing assay

Cells were seeded onto 6-well plates and incubated with high glucose and GSK343 as described above. After growing to 90% confluence, a sterile 20-μL micropipette tip was used to scratch the cell monolayers. Then, the cells were washed with PBS and supplemented with 2 mL of serum-free DMEM-F12. Images of migrating cells were obtained at 0 and 24 h as shown.

### 2.9. Transwell assay

Transwell cell culture inserts (pore size 8 μm; Corning Costar Corp.) were placed in DMEM-F12 supplemented with 15% fetal bovine serum in the lower compartment. HPMCs with different pretreatments were harvested with trypsin and resuspended in serum-free DMEM-F12. The upper chambers were seeded with 1 × 10^5^ cells/mL, which were allowed to attach at 37 °C for 12 h. Then, nonmigratory cells were removed from the upper surface of the chambers, and migrated cells in the lower membrane surface were fixed with 4% paraformaldehyde and stained with hematoxylin. Images of migrated cells were taken in 3 separate fields per membrane at 200× using phase contrast microscopy (Leica Microsystems) [28].

### 2.10. In vivo *experiments*

The experimental protocols for all animal studies were approved by the Animal Ethics Committee of Shandong Provincial Hospital Affiliated to Shandong First Medical University. Eight-week-old male Sprague–Dawley rats (130–140 g body weight, *n* = 24) were purchased from Shandong University Animal Center (Jinan, China). A rat peritoneal fibrosis model was generated by daily intraperitoneal (i.p.) injection of 4.25% peritoneal dialysis fluid (PDF) at 100 mL/kg for 4 weeks, combined with injections of lipopolysaccharide (LPS; 0.6 mg/kg) on Days 1, 3, 5, and 7 as described. Rats were randomly divided into three groups (*n* = 8/group) and treated as follows: Control, 20 mL i.p. normal saline (NS) daily; PDF, 4.25% PDF plus LPS i.p. as described above; and GSK343, the same treatment as the PDF group plus gavage administration of the EZH2 inhibitor. GSK343 was initiated from the second week just after the last injection of LPS. The period of GSK343 intervention lasted 3 weeks. Four weeks after the start of the experiment, the rats were euthanized. Both parietal and visceral peritoneal samples were harvested for regular histological evaluation and protein detection.

### 2.11. Histopathological and immunohistochemical analyses

Parietal peritoneal tissues were fixed in 10% neutral formalin and embedded in paraffin to obtain 3–4-μm-thick serial tissue sections. Deparaffinized sections were stained with hematoxylin and eosin (H&E) and Masson’s trichrome solution to analyze histopathological characteristics. Before immunohistochemistry, tissue sections were heated using citrate buffer (pH 6.0) to unmask antigens. Samples were pretreated with a 3% hydrogen peroxide solution to block endogenous peroxidase activity. Secondary goat anti-rat or anti-rabbit antibodies were applied as appropriate to detect the primary antibody. Primary antibodies were incubated overnight at 4 °C in a humidity chamber after blocking the slides for 30 min with 3% BSA. DAB (Dako) was used as an HRP substrate for signal detection [28].

### 2.12. Statistical analysis

Data are presented as the mean ± SD unless stated otherwise. One-way ANOVA was used to determine significant differences between groups. Dunnett’s test was used to perform multiple comparisons between groups. Two-tailed *p* values < .05 indicated statistical significance. All statistical analyses were performed using SPSS 20.0 software (SPSS Inc., Chicago, IL, USA).

## 3. Results

### 3.1. Ezh2 was upregulated and klotho was downregulated significantly in HPMCs treated with high glucose

**Figure 1.**
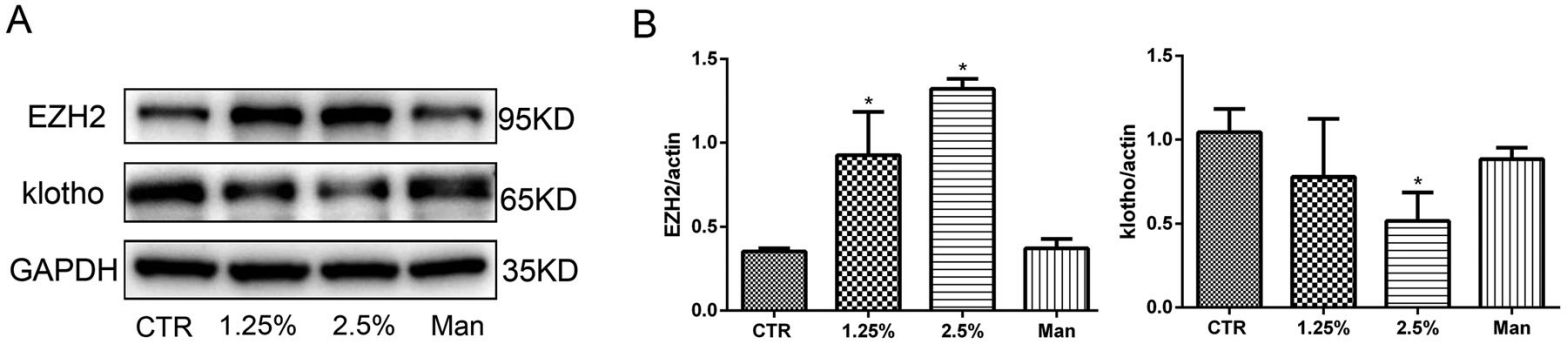
High glucose increases EZH2 expression and decreases klotho expression. (A) WB revealed that the expression of EZH2 was upregulated and that of klotho was downregulated in HPMCs stimulated with high glucose (HG). (B) Quantitative analysis of the data shown in A. (Data are the mean ± SD, *p < .05 vs. the control (CTR) group, *n* = 3.).

**Figure 2.**
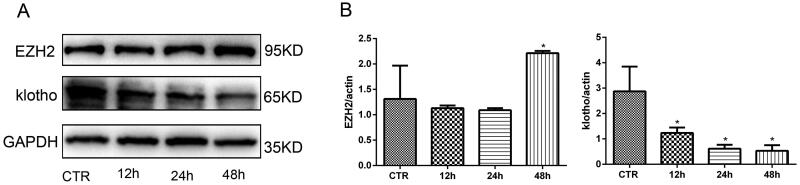
EZH2 was upregulated and klotho was downregulated significantly when HPMCs were stimulated with 2.5% HG for 48 h. (A) WB showed that high glucose increased the expression of EZH2 and decreased the expression of klotho in HPMCs when stimulated for 48 h. (B) Quantitative analysis of the data shown in A (Data are the mean ± SD, *p < .05 vs. the CTR group, *n* = 3).

To reveal whether EZH2 and klotho are involved in the pathogenesis of PD-related peritoneal fibrosis, we examined their expression in HPMCs treated with different concentrations of glucose for 48 h. The results showed that the expression of EZH2 was upregulated and the expression of klotho was downregulated in HPMCs incubated with high glucose in a concentration-dependent manner, while the high osmotic control was invalid (Figure 1(A,B)). Moreover, a time gradient (12, 24, 48 h) was also designed when the concentration of glucose was 2.5%. As shown in Figure 2(A,B), high glucose upregulated the expression of EZH2 and downregulated klotho in a time-dependent manner. Therefore, in the following experiments, 2.5% glucose concentration stimulation for 48 h was chosen.

### 3.2. Gsk343 alleviated EMT and fibrosis in high glucose-treated HPMCs

**Figure 3.**
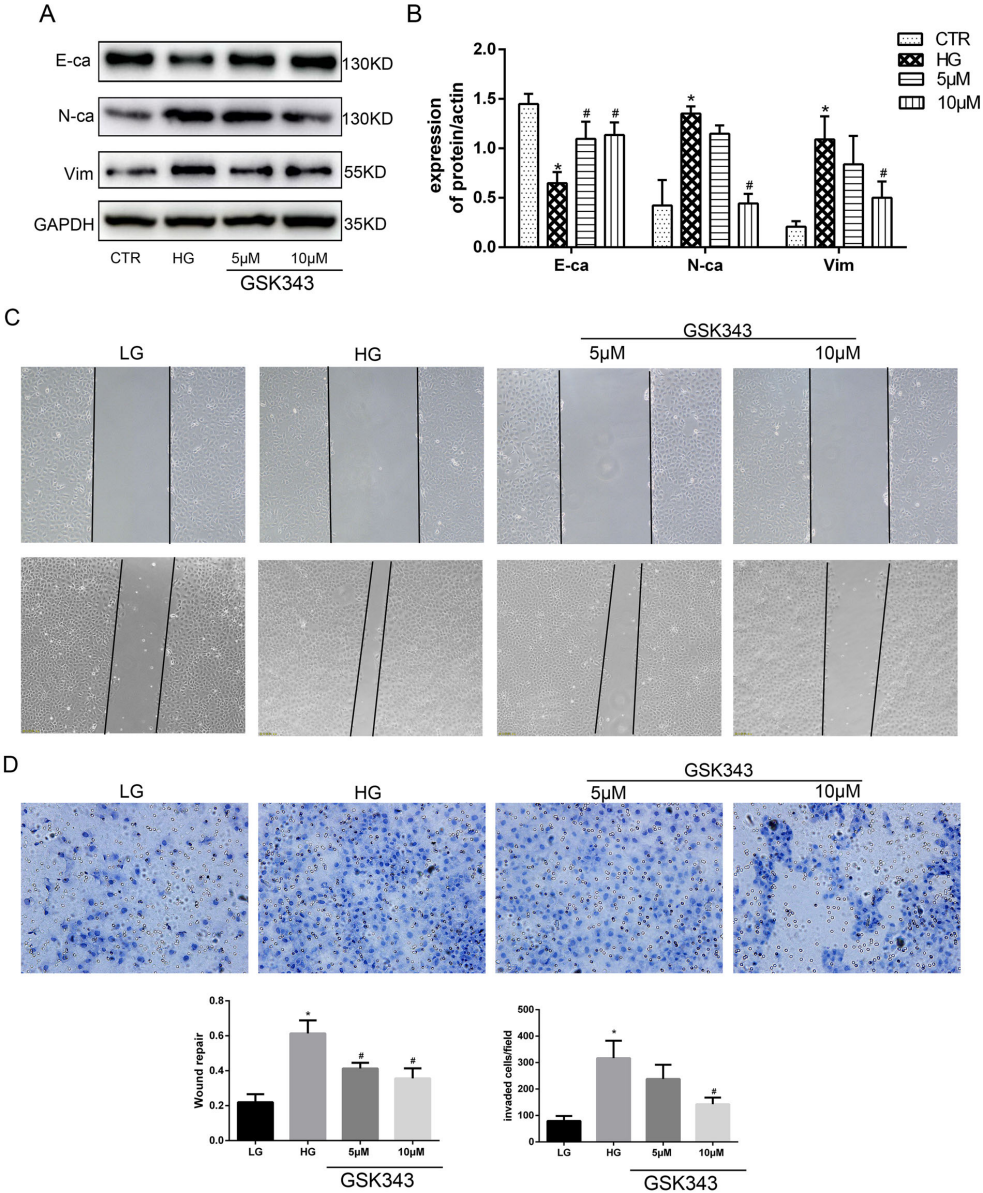
GSK343 mitigated high glucose-induced EMT and migration capacity. (A) The expression of E-cadherin, N-cadherin and vimentin was significantly reversed by GSK343 compared with that in the HG group when HPMCs were treated with high glucose in the presence or absence of GSK343. (B) Quantitative analysis of the data shown in A. (C) A wound-healing assay showed that GSK343 could inhibit the migration capacity induced by HG. (D) Transwell assays revealed that the high glucose-induced high migration capacity was relieved by GSK343. Statistical analysis of the results shown in Panels C and D was conducted (Data are the mean ± SD; *p < .05 vs. CTR group, #*p* < .05 vs. high glucose (HG) group, *n* = 3).

In our previous research, we proved the vital effect of EZH2 in high glucose-treated HPMCs. To further confirm the role of EZH2 in EMT and fibrosis in high glucose-treated HPMCs, a specific inhibitor of EZH2, GSK343, was added in the pretreatment with high glucose. According to previous research, 5 and 10 µM GSK343 were used to treat the cells [7]. As shown in Figure 3(A,B), WB revealed that GSK343 downregulated the high expression levels of N-cadherin and vimentin and upregulated the low expression level of E-cadherin induced by high glucose. Transwell experiments and wound healing assays also showed that GSK343 mitigated the cellular migration capacity induced by high glucose (Figure 39C, D)). In addition, we also concluded from the wound healing assay that high glucose induced a mesenchymal phenotype transition. As the cells became thin and slender, they resembled fibroblasts in the high glucose group, while cells in the low glucose and GSK343 treatment groups exhibited a cobblestone appearance.

### 3.3. Gsk343 reversed lipid deposition induced by high glucose

**Figure 4.**
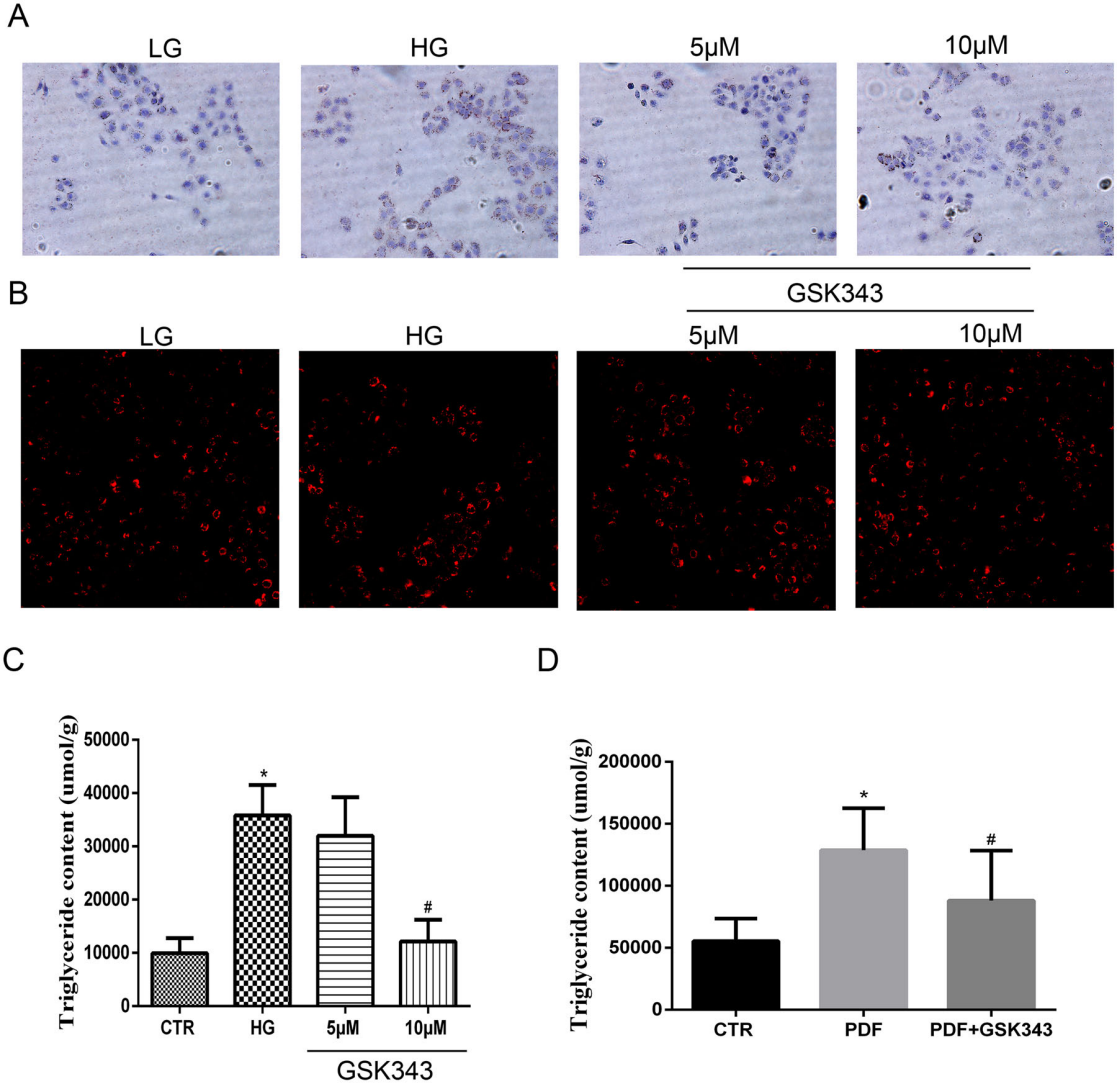
GSK343 relieved lipid deposition *in vitro* and *in vivo*. (A) Oil red O staining of HPMCs showed that high glucose-induced lipid accumulation could be attenuated by GSK343. (B) Nile red staining of HPMCs exhibited results similar to those demonstrated in Panel A. (C) TG quantitative determination of cells treated with high glucose in the presence or absence of GSK343 was conducted. The results showed that GSK343 could significantly reduce the TG content induced in the HG group. (D) The TG content of peritoneal tissue in different groups was detected by a TG detection assay kit. The results showed that GSK343 reduced the TG content compared with that in the PDF group (Data are the mean ± SD; *p < .05 vs. CTR group, #*p* < .05 vs. HG/PDF group, *n* = 3).

To further examine whether inhibition of EZH2 by GSK343 could alleviate lipid accumulation, HPMCs were exposed to high glucose with or without GSK343 pretreatment. As shown in Figure 4(A,B) Nile red and oil red O staining showed high glucose-induced accumulation of natural lipids, while GSK343 mitigated this accumulation. Quantitative TG content detection showed that the high TG content induced by high glucose was also decreased by GSK343, as shown in the panel of Figure 4(C). Additionally, *in vivo* experiments showed that GSK343 mitigated the TG content induced by PD, as shown in Figure 4(D). Overall, GSK343 reduced TG accumulation and lipid accumulation *in vivo* and *in vitro* in a PD model.

### 3.4. Gsk343 restored the downregulation of klotho induced by high glucose

**Figure 5.**
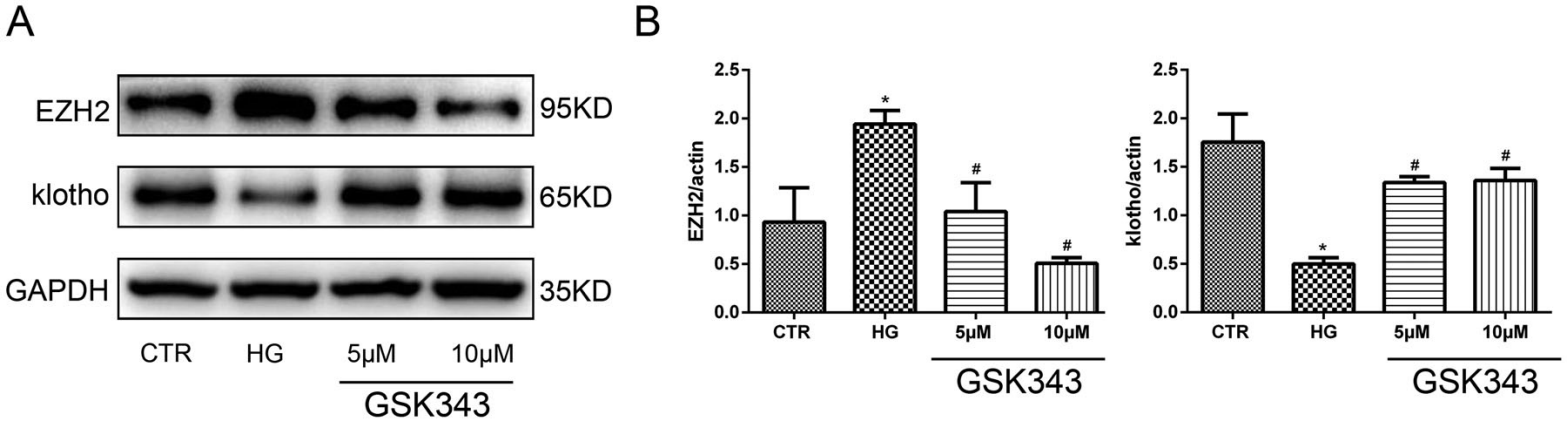
GSK343 restored the expression of EZH2 and klotho induced by high glucose. (A) The expression of EZH2 and klotho stimulated with high glucose in the presence or absence of GSK343 was detected by WB. The results showed that GSK343 could significantly reverse the expression of EZH2 and klotho compared with that in the HG group. (B) Quantitative analysis of the data shown in Panel A (Data are the mean ± SD; *p < .05 vs. CTR group, #*p* < .05 vs. HG group, *n* = 3).

To further clarify the relationship between EZH2 and klotho, we utilized GSK343. As shown in Figure 5(A,B), WB indicated that GSK343 could significantly restore the low expression level of klotho induced by high glucose. As the concentration increased, the effect of GSK343 became stronger. Therefore, we concluded that EZH2 is most likely located upstream of klotho.

### 3.5. Klotho mediated the protective effect of GSK343 by reducing lipid deposition

**Figure 6.**
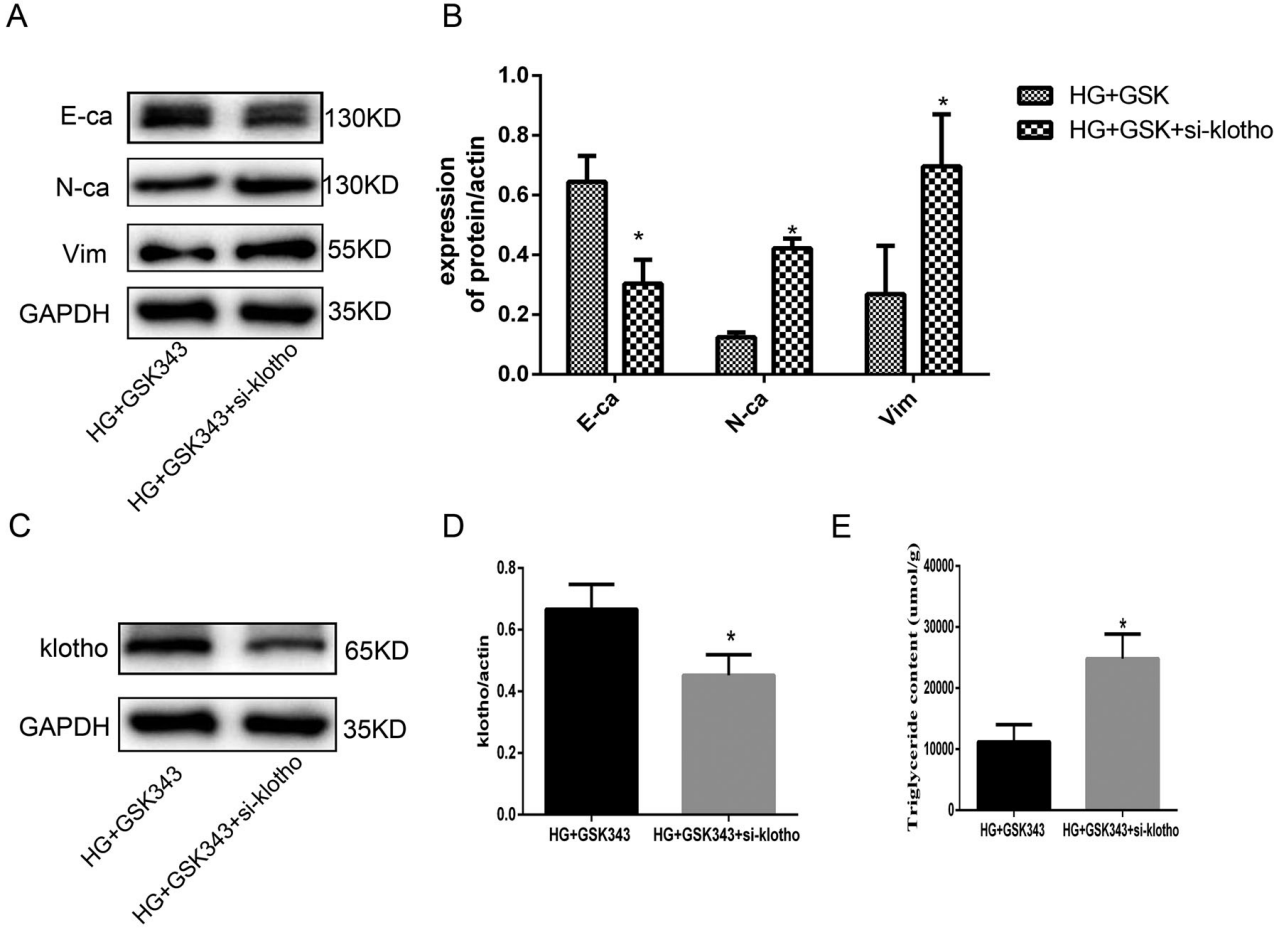
Rescue experiments showed that klotho mediated the protective effect of GSK343 on EMT and lipid accumulation. (A) WB revealed that the expression of E-cadherin, N-cadherin and vimentin was restored when HPMCs were treated with si-klotho compared with the HG + GSK343 group. (B) Quantitative analysis of the data shown in A. (C) WB revealed that the expression of klotho was restored when HPMCs were treated with si-klotho compared with the HG + GSK343 group. (D) Quantitative analysis of the data shown in C. (E) TG quantitative determination showed that the TG content was upregulated in the si-klotho + HG + GSK343 group compared with the HG + GSK343 group (Data are the mean ± SD; *p < .05 vs. HG + GSK343 group, *n* = 3).

We aimed to verify whether klotho mediated the effect of GSK343, and klotho rescue experiments were performed. Based on GSK343, we also applied si-klotho. As shown in Figure 6(A,B), WB demonstrated that EMT indices were partially restored by si-klotho. The upregulation of E-cadherin and downregulation of N-cadherin and vimentin were restored by si-klotho. Additionally, WB showed that the expression of klotho was significantly downregulated in the si-klotho group, which counteracted the upregulation of GSK343 (Figure 6(C,D)). Moreover, quantitative detection of TG content showed that the decreased content of TG due to GSK343 was elevated by si-klotho, as shown in Figure 6(E). Therefore, rescue experiments proved that klotho partially mediated the effect of GSK343.

### 3.6. *Gsk343 alleviated PD-related peritoneal fibrosis* via *klotho*

**Figure 7.**
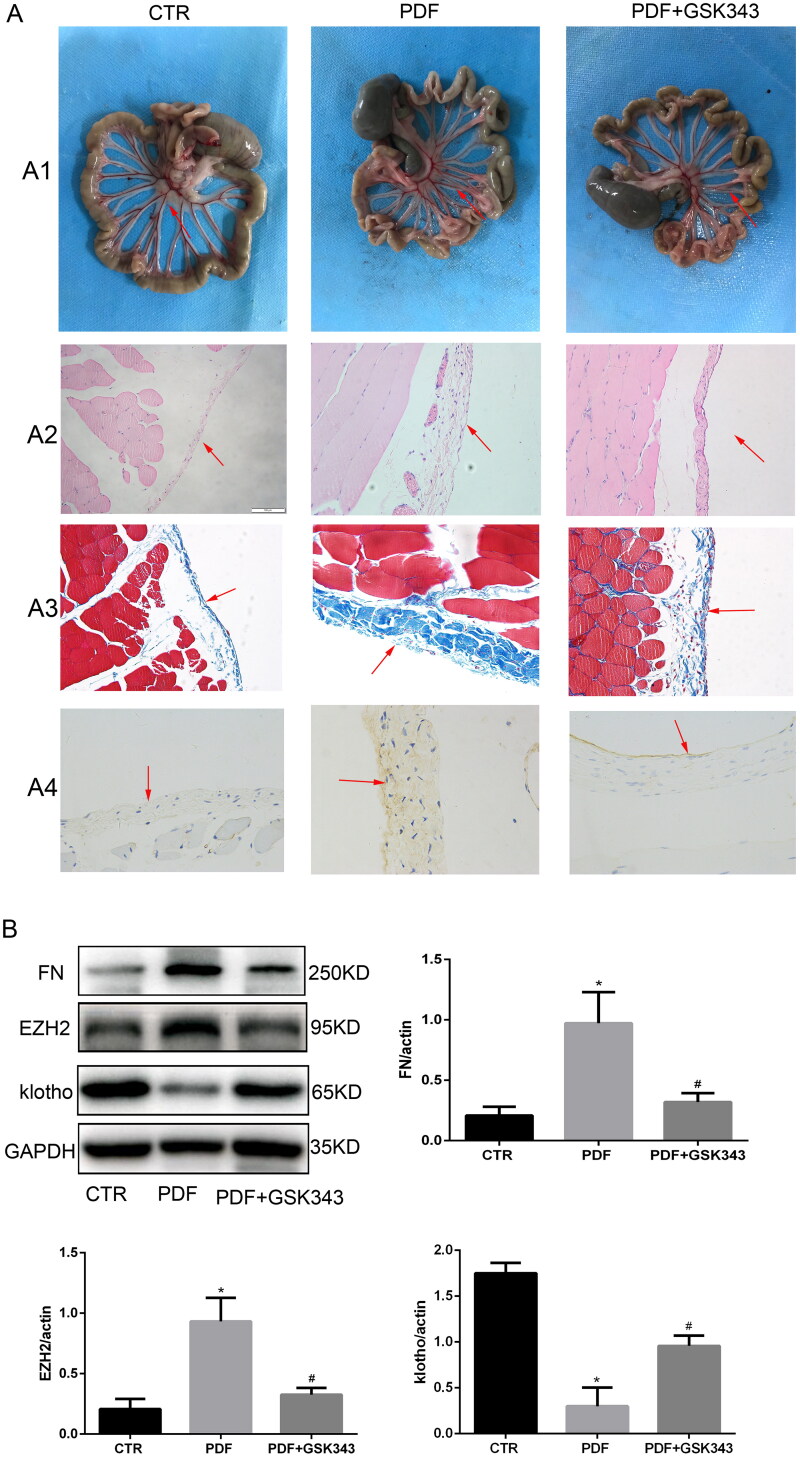
GSK343 alleviated PD-related peritoneal fibrosis and reversed the expression of klotho. (A) (A1) General images of the visceral peritoneum, (A2) HE staining of the parietal peritoneum, (A3) Masson staining of the parietal peritoneum and (A4) IHC of FN. All of the above results demonstrated that GSK343 could relieve peritoneal thickness, fibrosis and FN expression. (B) WB revealed that the expression of FN, EZH2 and klotho was significantly reversed by GSK343 compared with that in the PDF group (Data are the mean ± SD; *p < .05 vs. PDF group, *n* = 3).

To evaluate the inhibitory effects of GSK343 in PD-related peritoneal fibrosis, peritoneal thickness and fibrosis degree were assessed. In the *in vivo* experiment, we took a general picture of the peritoneum. The results of HE and Masson staining indicated that GSK343 could also alleviate PD-related peritoneal fibrosis, peritoneal thickness and angiogenesis in the rat model, as shown in Figure 7(A1-3). Specifically, the original monolayer of epithelial cells was markedly thickened, as shown by HE staining. In addition, the blue collagen fibers that were stained by Masson’s trichrome were also markedly increased in the PDF group. Combining these results, we could easily see that GSK343 could relieve the thickness and fibrosis that were induced in the PDF group. FN expression was assessed by immunohistochemistry and Western blotting in the peritoneum of rats with PD. WB and IHC indicated that it relieved the expression of FN induced by PD (Figure 7(A4,B)), in accordance with the results obtained *in vitro*. In addition, the results of *in vivo* experiments showed that GSK343 could upregulate the expression of klotho and downregulate the expression of EZH2, which were induced in the PD group. In summary, we can easily conclude that inhibition of EZH2 by GSK343 could reduce EMT and fibrosis.

## 4. Discussion

Long-term exposure to bioincompatible peritoneal dialysis fluid of the peritoneal membrane is a high-risk factor for peritoneal fibrosis, which, over time, can lead to peritoneal dysfunction. The core of peritoneal fibrosis is EMT and the inflammatory response, as demonstrated in previous literature [29]. Pathologically, the peritoneal membrane, which is continuously stimulated in nonphysiological peritoneal dialysis fluid, appears to be the phenotypic transition of PMCs, extracellular matrix deposition and cell shedding. Among all factors, high glucose stimulation to the surface of the peritoneal membrane and mesothelial cells is a rather important pathogenic factor [30]. In the current study, we observed that *in vitro* experiments, the expression of EZH2 was significantly upregulated, while the expression of klotho was significantly downregulated when HPMCs were treated with high glucose at various concentrations (1.25% or 2.5%). Additionally, in *in vivo* experiments, we found similar results in which EZH2 was upregulated and klotho was downregulated. After EZH2 was inhibited by the EZH2 inhibitor GSK343, lipid accumulation, EMT, and fibrosis were decreased, and the expression of klotho was significantly restored. These results were also replicated in *in vivo* experiments. These results further proved that EZH2 was a regulator of fibrosis and EMT, as reported previously [31], which was probably mediated by reducing lipid deposition. We hypothesize klotho may mediateyhe effect of GSK343. Mechanistically, through rescue experiments, we proved that the protective effect of GSK343 was rescued by si-klotho. Combining these findings, we found that EZH2 regulated lipid deposition, peritoneal fibrosis, and EMT mediated by klotho. Inhibition of klotho rescued the protective effect of GSK343. Overall, our study revealed that EZH2 plays a vital role in peritoneal fibrosis. To our knowledge, this is the first study to demonstrate the effect of the EZH2-klotho interaction on peritoneal lipotoxicity and fibrosis. This finding suggested EZH2 maybe a potential target for the treatment of peritoneal fibrosis.

Lipid deposits in nonfatty tissue (e.g., liver, skeleton muscle, heart) are called ectopic lipid deposits. Ectopic lipid accumulation, which causes tissue injury, is called lipotoxicity. In this process, high glucose plays a vital role. As a classical example, in diabetic nephropathy, high glucose per se eventually results in lipid metabolism dysfunction in podocytes according to numerous studies [32]. Additionally, high glucose could augment the incidence rate of nonalcoholic fatty liver disease [33]. Previous studies also revealed that lipid metabolism is involved in PD-related peritoneal fibrosis [17,18]. Abnormal lipid accumulation in normal tissue will cause a series of problems. Hence, targeting lipid metabolism has become a hotspot in current research. Reports have shown that statin treatment inhibits EMT changes in HG-treated HPMCs and PD rats [17,34]. These findings suggest that statins may be a promising therapeutic strategy for the preservation of peritoneal membrane integrity in long-term PD patients. In addition, a study demonstrated that PD-related peritoneal fibrosis was closely associated with increased accumulation of lipid droplets in the peritoneum 17,34. Inhibition of cellular lipid uptake and elevated cholesterol efflux are important aspects of the anti-fibrotic effects of rapamycin on PMCs [18]. Therefore, we can conclude that lipotoxicity mediates the progression of peritoneal fibrosis. In our study, we aimed to relieve lipotoxicity and reverse the progression of peritoneal fibrosis. Regarding how high glucose causes lipid accumulation, various experts have different opinions. Generally, lipids include triglycerides and cholesterol esters. Triglycerides primarily consist of free fatty acids (FFAs). The balance of FFAs is maintained by synthesis, transportation and metabolism. FFA influx is mediated by increased expression of the scavenger receptor platelet glycoprotein 4 (also known as CD36). Enhanced FFA *de novo* synthesis is mediated by SREBP-1C, FASN, and SCD-1, and weakened β-oxidation of FA is mediated by the rate-limiting enzyme CPT-1α. All these processes led to intracellular FFA accumulation. Cholesterol synthesis is controlled by HMGCR, and efflux is mediated by ABCA1 and ABCG1. However, in our current research, we did not reveal the detailed work; related research results will be published soon. However, we found that high glucose indeed induced lipid accumulation. GSK343 could relieve lipotoxicity and prevent peritoneal fibrosis, which are the primary findings in our initial study.

Klotho is an antiaging gene with both membrane-bound and soluble forms that was originally recognized in mice [19]. Subsequent large studies showed that it could suppress aging phenotypes and prolong lifespan when overexpressed. With the deepening of research, experts found that its deficiency leads to fibrosis in multiple organs, such as the kidney, heart, and lung. Fibrosis is also a characteristic feature of aging. Previously, in idiopathic pulmonary fibrosis and mouse pulmonary fibrosis models [35,36], klotho was found to decline, implying that it participated in pulmonary fibrosis. Recently, Huang also reported that klotho antagonizes pulmonary fibrosis by suppressing pulmonary fibroblast activation, migration, and extracellular matrix production [26]. Zhang performed *in vivo* and *in vitro* studies to assess the effects of klotho administration on acute lung injury induced by paraquat and demonstrated that klotho had a protective effect on pulmonary epithelial cell damage, significantly improved the survival rate and alleviated lung tissue fibrosis [37]. In addition, Ding reported that klotho inhibits angiotensin II-induced cardiac hypertrophy, fibrosis, and dysfunction in mice through suppression of the transforming growth factor-β1 signaling pathway [38]. In a PD model, the peritoneal membrane was protected from peritoneal fibrosis in klotho transgenic rats through attenuation of the Wnt/β-catenin signaling pathway, but the mechanism was not shown [27]. In addition, klotho is a key molecule in regulating lipid metabolism in the liver [39]. In our research, we found that the expression of klotho was significantly decreased in high glucose induced HPMCs. Inhibition of EZH2 could restore it which means klotho located downstream of EZH2. Moreover, rescue experiments further verified that klotho could rescue the effect of gsk343. Therefore we speculated that klotho was involved in peritoneal fibrosis possibly mediated by regulating lipid metabolism. However the detailed mechanism of EZH2 and klotho in peritoneal fibrosis remained unclear. Further researches were needed to reveal.

EZH2, a key molecule highlighted in recent research, is of great value in the field of cancer. The vital effect of EZH2 in PD-related peritoneal fibrosis has been expounded on in our previous research. In the present study, we found that it can relieve peritoneal lipotoxicity mediated by klotho, while its related mechanism was not elucidated. EZH2 may also be involved in the regulation of klotho expression through epigenetic modification. Further research is needed.

In conclusion, our findings confirm that GSK343 protects against EMT and fibrosis induced by high glucose medium. Besides the earlier demonstration of its inhibition of EZH2 and its attenuation of in TGF-β gene transcription, our experiments demonstrate that protection against PD fibrosis may be mediated by reducing lipid deposition *via* klotho expression. Based on the current results, targeting EZH2 is extremely promising .
